# 1,4,10,13-Tetra­oxa-7,16-diazo­niacyclo­octadecane bis­[tetra­chloridoaurate(III)] dihydrate

**DOI:** 10.1107/S1600536808015353

**Published:** 2008-05-24

**Authors:** Leila Hojjat Kashani, Mohammad Yousefi, Vahid Amani, Hamid Reza Khavasi

**Affiliations:** a Islamic Azad University, Shahr-e-Rey Branch, Tehran, Iran; b Department of Chemistry, Shahid Beheshti University, Tehran 1983963113, Iran

## Abstract

The asymmetric unit of the title compound, (C_12_H_28_N_2_O_4_)[AuCl_4_]_2_·2H_2_O, contains one half-cation, one anion and one water mol­ecule; the cation is centrosymmetric. The Au ion has a square-planar coordination. In the crystal structure, intra­molecular N—H⋯O and O—H⋯O, and inter­molecular N—H⋯O, O—H⋯Cl and N—H⋯Cl hydrogen bonds link the ions and water mol­ecules, forming a supra­molecular structure.

## Related literature

For related literature, see: Calleja *et al.* (2001 ▶); Chekhlov (2000 ▶, 2001 ▶, 2005 ▶); Chekhlov & Martynov (1998 ▶); Chekhlov *et al.* (1994 ▶); Fonari *et al.* (2004 ▶); Hasan *et al.* (1999 ▶); Johnson & Steed (1998 ▶); Moers *et al.* (2000 ▶); Simonov *et al.* (2003 ▶); Yap *et al.* (1995 ▶); Yousefi, Amani & Khavasi (2007 ▶); Yousefi, Teimouri *et al.* (2007 ▶); Zhang *et al.* (2006 ▶).
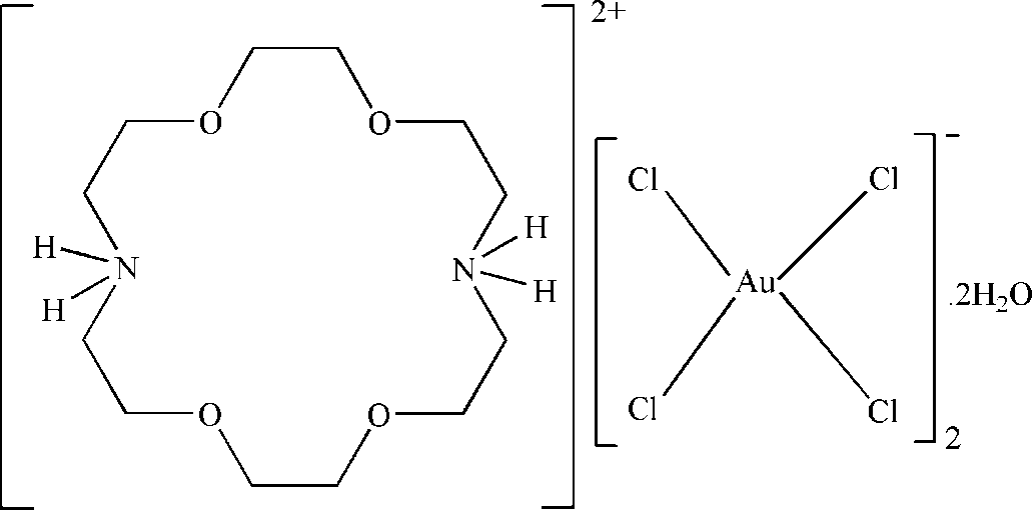


## Experimental

### 

#### Crystal data

(C_12_H_28_N_2_O_4_)[AuCl_4_]_2_·2H_2_O
*M* *_r_* = 977.94
Triclinic, 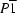

*a* = 8.0168 (10) Å
*b* = 8.3359 (9) Å
*c* = 11.2989 (15) Å
α = 73.063 (11)°
β = 75.965 (10)°
γ = 74.929 (9)°
*V* = 686.02 (15) Å^3^
*Z* = 1
Mo *K*α radiation
μ = 11.49 mm^−1^
*T* = 120 (2) K
0.32 × 0.22 × 0.20 mm

#### Data collection

Stoe IPDSII diffractometer
Absorption correction: numerical  (*X-SHAPE* and *X-RED*; Stoe & Cie, 2005 ▶)*T*_min_ = 0.065, *T*_max_ = 0.108
4188 measured reflections
2390 independent reflections
2381 reflections with *I* > 2σ(*I*)
*R*_int_ = 0.027

#### Refinement

*R*[*F* ^2^ > 2σ(*F* ^2^)] = 0.017
*wR*(*F* ^2^) = 0.045
*S* = 1.18
2390 reflections
144 parameters
H atoms treated by a mixture of independent and constrained refinement
Δρ_max_ = 0.59 e Å^−3^
Δρ_min_ = −0.67 e Å^−3^

### 

Data collection: *X-AREA* (Stoe & Cie, 2005 ▶); cell refinement: *X-AREA*; data reduction: *X-RED* (Stoe & Cie, 2005 ▶); program(s) used to solve structure: *SHELXS97* (Sheldrick, 2008 ▶); program(s) used to refine structure: *SHELXL97* (Sheldrick, 2008 ▶); molecular graphics: *ORTEP-3 for Windows* (Farrugia, 1997 ▶); software used to prepare material for publication: *WinGX* (Farrugia, 1999 ▶).

## Supplementary Material

Crystal structure: contains datablocks I, global. DOI: 10.1107/S1600536808015353/hk2465sup1.cif

Structure factors: contains datablocks I. DOI: 10.1107/S1600536808015353/hk2465Isup2.hkl

Additional supplementary materials:  crystallographic information; 3D view; checkCIF report

## Figures and Tables

**Table:** 

Cl1—Au1	2.2796 (11)
Cl2—Au1	2.2877 (10)
Cl3—Au1	2.2912 (11)
Cl4—Au1	2.2751 (11)

**Table:** 

Cl4—Au1—Cl1	90.20 (4)
Cl4—Au1—Cl2	176.52 (4)
Cl1—Au1—Cl2	89.96 (4)
Cl4—Au1—Cl3	90.30 (4)
Cl1—Au1—Cl3	176.79 (3)
Cl2—Au1—Cl3	89.74 (4)

**Table 2:** Hydrogen-bond geometry (Å, °)

*D*—H⋯*A*	*D*—H	H⋯*A*	*D*⋯*A*	*D*—H⋯*A*
N1—H1*C*⋯O1^i^	0.90	2.49	2.791 (5)	100
N1—H1*C*⋯O3	0.90	1.98	2.844 (3)	160
N1—H1*D*⋯Cl1^ii^	0.90	2.81	3.540 (4)	139
N1—H1*D*⋯Cl2^ii^	0.90	2.49	3.262 (3)	143
O3—H3*C*⋯O1	0.76 (6)	2.14 (6)	2.858 (4)	158 (6)
O3—H3*C*⋯O2	0.76 (6)	2.51 (6)	3.057 (3)	130 (5)
O3—H3*D*⋯Cl3^iii^	0.81 (7)	2.59 (6)	3.378 (4)	167.00

Symmetry codes: (i) 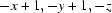; (ii) 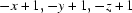; (iii) 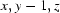.

**Fig. 1.**
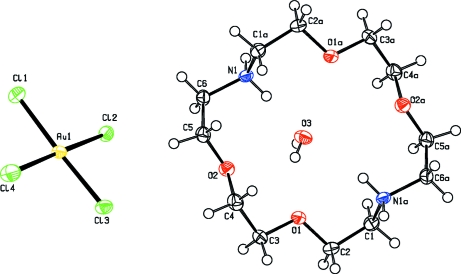
The asymmetric unit of the title molecule, with the atom-numbering scheme. Displacement ellipsoids are drawn at the 50% probability level [symmetry code: (a) 1 - x, 1 - y, -z].

**Fig. 2.**
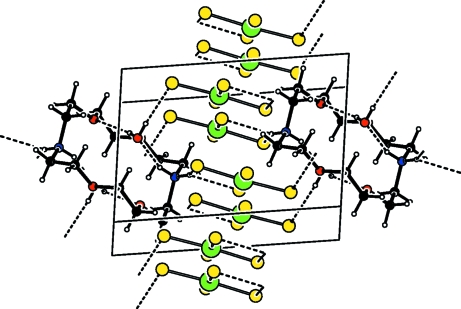
A partial packing diagram of (I). Hydrogen bonds are shown as dashed lines.

## supplementary crystallographic information

### Comment

Recently, we reported the synthesis and crystal structure of the [(H_2_DA18C6)Cl_2_], (II), (Yousefi, Amani & Khavasi, 2007) and [(H_2_DA18C6)][PtCl_6_].2H_2_O, (III), (Yousefi, Teimouri *et al.*, 2007) [where H_2_DA18C6 is 1,10-Diazonia-18-crown-6]. Several proton transfer systems using 1,10-diaza-18-crown-6, with proton donor molecules, such as [(H_2_DA18C6)I_2_.2H_2_O], (IV), (Chekhlov, 2005), [(H_2_DA18C6)(C_2_HO_4_)_2_], (V), and [(H_2_DA18C6)_2_(C_2_O_4_)_2_.2H_2_O], (VI), (Chekhlov, 2000), [(H_2_DA18C6)(picrate)_2_], (VII), (Chekhlov, 2001), [(H_2_DA18C6)(HPTD)_2_], (VIII), (Simonov *et al.*, 2003), [(H_2_DA18C6)(PD)_2_.(H_2_O)_4_], (IX), and [(H_2_DA18C6)(PS)_2_.(H_2_O)_2_], (*X*), (Fonari *et al.*, 2004), [(H_2_DA18C6)(CCl_3_COO)_2_(CCl_3_COOH)_2_], (XI), (Chekhlov *et al.*, 1994), [(H_2_DA18C6)(CCl_3_COO)_2_], (XII), (Chekhlov & Martynov, 1998), and {[H_2_DA18C6][(ArSO_2_)_2_N]_2_}, (XIII), (Moers *et al.*, 2000) [where C_2_O_4_ is oxalate, HPTD is (4*Z*,5E)-pyrimidine-2,4,5,6(1*H*,3H) -tetraone 4,5-dioxime anion, PD is 2-(2-methylphenyl)-2*H*-[1,2,3]- triazolo[4,5-*d*]pyrimidine-5,7(4*H*,6H)-dione 3-oxide anion, PS is 6-amino-2,4-dioxo-1,2,3,4-tetrahydropyrimidin-5-ylsulfamate and (ArSO_2_)_2_N is bis(4-chlorobenzenesulfonyl)imide] have been synthesized and characterized by single-crystal X-ray diffraction methods.

There are also several proton transfer systems using HAuCl_4_ with proton acceptor molecules, such as [EMI][AuCl_4_] (XIV) and [BMI]_2_[AuCl_4_].2H_2_O, (XV), (Hasan *et al.*, 1999), [H_2_bipy][AuCl_4_][Cl], (XVI), (Zhang *et al.*, 2006), [H_7_O_3_][15-crown-5][AuCl_4_], (XVII) and [H_5_O_2_][benzo-15-crown-5] _2_[AuCl_4_], (XVIII), (Johnson & Steed, 1998), [H_5_O_2_]_2_[12-crown-4]_2_ [AuCl_4_]_2_, (XIX), [H_3_O][18-crown-6][AuCl_4_], (XX) and [H_3_O] [4-nitrobenzo-18-crown-6][AuCl_4_], (XXI), (Calleja *et al.*, 2001) and [DPpy.H][AuCl_4_], (XXII), (Yap *et al.*, 1995) [where EMI is 1-ethyl-3-methylimidazolium, BMI is 1-butyl-3-methylimidazolium, H_2_bipy is 2,2'-bipyridinium and DPpy.H is 2,6-Diphenylpyridinium] have been synthesized and characterized by single-crystal X-ray diffraction methods. We report herein the synthesis and crystal structure of the title compound, (I).

The asymmetric unit of (I), (Fig. 1) contains one half-cation, one anion and one water molecule; the cation is centrosymmetric. The Au ion has a square-planar coordination (Table 1). The bond lengths and angles, in cation, are in good agreement with the corresponding values in (II), (III) and (IV). Also, the Au-Cl bond lengths and angles (Table 1) are within normal range [XXII].

In the crystal structure, intramolecular N-H···O and O-H···O and intermolecular N-H···O, O-H···Cl and N-H···Cl hydrogen bonds (Table 2) link the molecules to form a supramolecular structure (Fig. 2), in which they may be effective in the stabilization of the structure.

### Experimental

For the preparation of the title compound, (I), a solution of 1,10-diaza-18 -crown-6 (0.10 g, 0.37 mmol) in EtOH (20 ml) was added to a solution of HAuCl_4_.3H_2_O, (0.29 g, 0.74 mmol) in water (30 ml) and the resulting yellow solution was stirred for 10 min at 313 K. Then, it was left to evaporate slowly at room temperature. After one week, yellow prismatic crystals of (I) were isolated (yield; 0.26 g; 72.0%).

### Refinement

H atoms (for H_2_O) were located in a difference syntheses and refined [O-H = 0.71 (6) and 0.76 (6) Å; U_iso_(H) = 0.019 (15) and 0.034 (17) Å^2^]. The remaining H atoms were positioned geometrically, with N-H = 0.90 Å (for NH_2_) and C-H = 0.97 Å for methylene H and constrained to ride on their parent atoms with U_iso_(H) = 1.2U_eq_(C,N).

### Crystal data

**Table:**

(C_12_H_28_N_2_O_4_)[AuCl_4_]_2_·2H_2_O	*Z* = 1
*M**_r_* = 977.94	*F*_000_ = 460
Triclinic, *P*1	*D*_x_ = 2.367 Mg m^−^^3^
Hall symbol: -P 1	Mo *K*α radiation λ = 0.71073 Å
*a* = 8.0168 (10) Å	Cell parameters from 1139 reflections
*b* = 8.3359 (9) Å	θ = 1.9–25.2º
*c* = 11.2989 (15) Å	µ = 11.49 mm^−^^1^
α = 73.063 (11)º	*T* = 120 (2) K
β = 75.965 (10)º	Block, yellow
γ = 74.929 (9)º	0.32 × 0.22 × 0.20 mm
*V* = 686.02 (15) Å^3^	

### Data collection

**Table:**

Stoe IPDSII diffractometer	2390 independent reflections
Radiation source: fine-focus sealed tube	2381 reflections with *I* > 2σ(*I*)
Monochromator: graphite	*R*_int_ = 0.027
Detector resolution: 0.15 mm pixels mm^-1^	θ_max_ = 25.2º
*T* = 120(2) K	θ_min_ = 1.9º
rotation method scans	*h* = −9→8
Absorption correction: numericalshape of crystal determined optically	*k* = −9→8
*T*_min_ = 0.065, *T*_max_ = 0.108	*l* = −12→12
4188 measured reflections	

### Refinement

**Table:**

Refinement on *F*^2^	Secondary atom site location: difference Fourier map
Least-squares matrix: full	Hydrogen site location: inferred from neighbouring sites
*R*[*F*^2^ > 2σ(*F*^2^)] = 0.017	H atoms treated by a mixture of independent and constrained refinement
*wR*(*F*^2^) = 0.045	*w* = 1/[σ^2^(*F*_o_^2^) + (0.0216*P*)^2^ + 1.1824*P*] where *P* = (*F*_o_^2^ + 2*F*_c_^2^)/3
*S* = 1.18	(Δ/σ)_max_ = 0.018
2390 reflections	Δρ_max_ = 0.59 e Å^−^^3^
144 parameters	Δρ_min_ = −0.67 e Å^−^^3^
Primary atom site location: structure-invariant direct methods	Extinction correction: none

### Special details

**Table:**

Experimental. (X-SHAPE and X-RED; Stoe & Cie, 2005)
Geometry. All e.s.d.'s (except the e.s.d. in the dihedral angle between two l.s. planes) are estimated using the full covariance matrix. The cell e.s.d.'s are taken into account individually in the estimation of e.s.d.'s in distances, angles and torsion angles; correlations between e.s.d.'s in cell parameters are only used when they are defined by crystal symmetry. An approximate (isotropic) treatment of cell e.s.d.'s is used for estimating e.s.d.'s involving l.s. planes.
Refinement. Refinement of F^2^ against ALL reflections. The weighted R-factor wR and goodness of fit S are based on F^2^, conventional R-factors R are based on F, with F set to zero for negative F^2^. The threshold expression of F^2^ > 2sigma(F^2^) is used only for calculating R-factors(gt) etc. and is not relevant to the choice of reflections for refinement. R-factors based on F^2^ are statistically about twice as large as those based on F, and R- factors based on ALL data will be even larger.

### Fractional atomic coordinates and isotropic or equivalent isotropic displacement parameters (Å^2^)

**Table:**

	*x*	*y*	*z*	*U*_iso_*/*U*_eq_	
Au1	0.774200 (17)	0.949008 (17)	0.436516 (13)	0.01917 (8)	
Cl1	0.65158 (14)	0.91699 (13)	0.64419 (10)	0.0271 (2)	
Cl2	0.77516 (14)	0.66859 (13)	0.45325 (10)	0.0249 (2)	
Cl3	0.88196 (14)	0.98407 (13)	0.22535 (10)	0.0257 (2)	
Cl4	0.79013 (16)	1.22269 (14)	0.42240 (12)	0.0362 (3)	
O1	0.8055 (4)	0.5576 (4)	−0.1123 (3)	0.0230 (6)	
O2	0.6857 (4)	0.6728 (3)	0.1006 (3)	0.0227 (6)	
O3	0.6136 (4)	0.3371 (5)	0.0862 (4)	0.0259 (7)	
H3C	0.666 (7)	0.406 (7)	0.049 (5)	0.019 (15)*	
H3D	0.689 (8)	0.264 (8)	0.118 (5)	0.034 (17)*	
N1	0.3824 (4)	0.5189 (4)	0.2598 (3)	0.0193 (7)	
H1C	0.4424	0.4823	0.1909	0.023*	
H1D	0.3672	0.4259	0.3230	0.023*	
C1	0.7938 (5)	0.3778 (5)	−0.2343 (4)	0.0231 (9)	
H1A	0.7787	0.2856	−0.1594	0.028*	
H1B	0.8544	0.3272	−0.3043	0.028*	
C2	0.9019 (5)	0.4874 (5)	−0.2162 (4)	0.0228 (9)	
H2A	1.0152	0.4194	−0.1983	0.027*	
H2B	0.9210	0.5783	−0.2913	0.027*	
C3	0.8743 (5)	0.6921 (5)	−0.0984 (4)	0.0232 (9)	
H3A	0.9062	0.7677	−0.1798	0.028*	
H3B	0.9779	0.6447	−0.0597	0.028*	
C4	0.7321 (6)	0.7883 (5)	−0.0161 (4)	0.0259 (9)	
H4A	0.7733	0.8800	−0.0025	0.031*	
H4B	0.6303	0.8385	−0.0566	0.031*	
C5	0.5532 (5)	0.7528 (5)	0.1851 (4)	0.0242 (9)	
H5A	0.4568	0.8215	0.1431	0.029*	
H5B	0.5998	0.8274	0.2147	0.029*	
C6	0.4895 (5)	0.6163 (5)	0.2940 (4)	0.0232 (9)	
H6A	0.4190	0.6689	0.3610	0.028*	
H6B	0.5900	0.5367	0.3257	0.028*	

### Atomic displacement parameters (Å^2^)

**Table:**

	*U*^11^	*U*^22^	*U*^33^	*U*^12^	*U*^13^	*U*^23^
Au1	0.01758 (11)	0.01715 (11)	0.02133 (12)	−0.00433 (7)	−0.00015 (7)	−0.00455 (7)
Cl1	0.0296 (5)	0.0268 (5)	0.0227 (6)	−0.0041 (4)	−0.0003 (4)	−0.0077 (4)
Cl2	0.0334 (6)	0.0204 (5)	0.0217 (5)	−0.0106 (4)	−0.0006 (4)	−0.0055 (4)
Cl3	0.0271 (5)	0.0229 (5)	0.0236 (5)	−0.0074 (4)	0.0019 (4)	−0.0037 (4)
Cl4	0.0433 (7)	0.0205 (5)	0.0411 (7)	−0.0105 (5)	0.0085 (5)	−0.0118 (5)
O1	0.0223 (14)	0.0256 (15)	0.0229 (16)	−0.0110 (12)	0.0022 (12)	−0.0083 (12)
O2	0.0249 (15)	0.0196 (14)	0.0227 (16)	−0.0063 (12)	−0.0022 (12)	−0.0039 (12)
O3	0.0223 (17)	0.0229 (17)	0.0312 (19)	−0.0092 (16)	0.0008 (15)	−0.0049 (14)
N1	0.0197 (17)	0.0206 (17)	0.0162 (17)	−0.0061 (13)	0.0016 (14)	−0.0046 (14)
C1	0.020 (2)	0.023 (2)	0.027 (2)	−0.0009 (16)	−0.0022 (17)	−0.0108 (18)
C2	0.018 (2)	0.027 (2)	0.022 (2)	−0.0043 (17)	0.0019 (17)	−0.0077 (18)
C3	0.023 (2)	0.024 (2)	0.025 (2)	−0.0102 (17)	−0.0032 (17)	−0.0057 (18)
C4	0.032 (2)	0.019 (2)	0.027 (2)	−0.0099 (18)	−0.0062 (19)	−0.0021 (17)
C5	0.025 (2)	0.021 (2)	0.029 (2)	−0.0057 (17)	−0.0029 (18)	−0.0100 (18)
C6	0.022 (2)	0.026 (2)	0.026 (2)	−0.0046 (17)	−0.0044 (17)	−0.0123 (18)

### Geometric parameters (Å, °)

**Table:**

Cl1—Au1	2.2796 (11)	C2—H2B	0.9700
Cl2—Au1	2.2877 (10)	C3—O1	1.432 (5)
Cl3—Au1	2.2912 (11)	C3—C4	1.500 (6)
Cl4—Au1	2.2751 (11)	C3—H3A	0.9700
O3—H3C	0.71 (6)	C3—H3B	0.9700
O3—H3D	0.76 (6)	C4—O2	1.419 (5)
N1—C1^i^	1.496 (5)	C4—H4A	0.9700
N1—H1C	0.9000	C4—H4B	0.9700
N1—H1D	0.9000	C5—O2	1.412 (5)
C1—C2	1.495 (6)	C5—C6	1.501 (6)
C1—N1^i^	1.496 (5)	C5—H5A	0.9700
C1—H1A	0.9700	C5—H5B	0.9700
C1—H1B	0.9700	C6—N1	1.501 (5)
C2—O1	1.429 (5)	C6—H6A	0.9700
C2—H2A	0.9700	C6—H6B	0.9700
			
Cl4—Au1—Cl1	90.20 (4)	H2A—C2—H2B	108.6
Cl4—Au1—Cl2	176.52 (4)	O1—C3—C4	106.8 (3)
Cl1—Au1—Cl2	89.96 (4)	O1—C3—H3A	110.4
Cl4—Au1—Cl3	90.30 (4)	C4—C3—H3A	110.4
Cl1—Au1—Cl3	176.79 (3)	O1—C3—H3B	110.4
Cl2—Au1—Cl3	89.74 (4)	C4—C3—H3B	110.4
C2—O1—C3	113.3 (3)	H3A—C3—H3B	108.6
C5—O2—C4	112.8 (3)	O2—C4—C3	108.8 (3)
H3C—O3—H3D	103 (6)	O2—C4—H4A	109.9
C1^i^—N1—C6	113.5 (3)	C3—C4—H4A	109.9
C1^i^—N1—H1C	108.9	O2—C4—H4B	109.9
C6—N1—H1C	108.9	C3—C4—H4B	109.9
C1^i^—N1—H1D	108.9	H4A—C4—H4B	108.3
C6—N1—H1D	108.9	O2—C5—C6	108.5 (3)
H1C—N1—H1D	107.7	O2—C5—H5A	110.0
C2—C1—N1^i^	110.8 (3)	C6—C5—H5A	110.0
C2—C1—H1A	109.5	O2—C5—H5B	110.0
N1^i^—C1—H1A	109.5	C6—C5—H5B	110.0
C2—C1—H1B	109.5	H5A—C5—H5B	108.4
N1^i^—C1—H1B	109.5	C5—C6—N1	112.9 (3)
H1A—C1—H1B	108.1	C5—C6—H6A	109.0
O1—C2—C1	106.6 (3)	N1—C6—H6A	109.0
O1—C2—H2A	110.4	C5—C6—H6B	109.0
C1—C2—H2A	110.4	N1—C6—H6B	109.0
O1—C2—H2B	110.4	H6A—C6—H6B	107.8
C1—C2—H2B	110.4		
			
N1^i^—C1—C2—O1	59.1 (4)	C1—C2—O1—C3	−168.3 (3)
O1—C3—C4—O2	58.5 (4)	C4—C3—O1—C2	162.2 (3)
O2—C5—C6—N1	−71.6 (4)	C6—C5—O2—C4	169.1 (3)
C5—C6—N1—C1^i^	−70.3 (4)	C3—C4—O2—C5	179.4 (3)

Symmetry codes: (i) −*x*+1, −*y*+1, −*z*.

### Hydrogen-bond geometry (Å, °)

**Table:**

*D*—H···*A*	*D*—H	H···*A*	*D*···*A*	*D*—H···*A*
N1—H1C···O1^i^	0.90	2.49	2.791 (5)	100
N1—H1C···O3	0.90	1.98	2.844 (3)	160
N1—H1D···Cl1^ii^	0.90	2.81	3.540 (4)	139
N1—H1D···Cl2^ii^	0.90	2.49	3.262 (3)	143
O3—H3C···O1	0.76 (6)	2.14 (6)	2.858 (4)	158 (6)
O3—H3C···O2	0.76 (6)	2.51 (6)	3.057 (3)	130 (5)
O3—H3D···Cl3^iii^	0.81 (7)	2.59 (6)	3.378 (4)	167.00

Symmetry codes: (i) −*x*+1, −*y*+1, −*z*; (ii) −*x*+1, −*y*+1, −*z*+1; (iii) *x*, *y*−1, *z*.
